# CROSS-CULTURAL COMPARISON OF A LATENT DEMENTIA INDEX IN MEXICO AND THE UNITED STATES

**DOI:** 10.1093/geroni/igac059.2817

**Published:** 2022-12-20

**Authors:** Joseph Saenz, Christopher Beam

**Affiliations:** University of Southern California, Los Angeles, California, United States; University of Southern California, Los Angeles, California, United States

## Abstract

Latent variable models have recently been used to extract latent dementia index scores using cognitive and functional ability data. Yet, no cross-cultural comparisons have been conducted. This analysis tests metric measurement invariance of a latent dementia indicator (LDI) among older adults in Mexico and the US using different statistical criteria. We then test whether demographic risk factors relate with the LDI similarly depending on criteria used to test measurement invariance. Data included the MexCog (Mexico, n=2,265) and Harmonized Cognitive Assessment Protocol (US HCAP, n=3,347). The LDI is a latent variable measured using 13 cognitive and 10 functional common items. Measurement invariance was tested in multiple group confirmatory factor analyses using both nested chi-square difference tests and comparisons of comparative fit indices (CFI). Covariances between demographic risk factors (age and education) and the LDI were compared across models achieved using different measurement invariance criteria. Full metric invariance of the LDI was achieved using CFI comparisons but not with chi-square comparisons, which required nine loadings to be freely estimated across studies to achieve partial metric invariance. Regardless of criteria used to test measurement invariance, age and education correlated with the LDI in both studies in expected directions with similar parameter estimates regardless of approach used to test measurement invariance. The LDI may be a valid tool to compare associations between risk factors and dementia in the HCAP and MexCog. The LDI related with demographic factors in expected ways and findings were robust to statistical approach used for measurement invariance testing.

